# Histone Deacetylase Inhibitors in Pediatric Brain Cancers: Biological Activities and Therapeutic Potential

**DOI:** 10.3389/fcell.2020.00546

**Published:** 2020-07-10

**Authors:** Alexandre Perla, Lívia Fratini, Paula S. Cardoso, Carolina Nör, André T. Brunetto, Algemir L. Brunetto, Caroline Brunetto de Farias, Mariane Jaeger, Rafael Roesler

**Affiliations:** ^1^Cancer and Neurobiology Laboratory, Experimental Research Center, Clinical Hospital (CPE-HCPA), Federal University of Rio Grande do Sul, Porto Alegre, Brazil; ^2^Department of Pharmacology, Institute for Basic Health Sciences, Federal University of Rio Grande do Sul, Porto Alegre, Brazil; ^3^The Arthur and Sonia Labatt Brain Tumour Research Centre, The Hospital for Sick Children, Toronto, ON, Canada; ^4^Developmental and Stem Cell Biology Program, The Hospital for Sick Children, Toronto, ON, Canada; ^5^Children’s Cancer Institute, Porto Alegre, Brazil

**Keywords:** medulloblastoma, ependymoma, glioma, histone deacetylase inhibitors, epigenetics, brain tumor

## Abstract

Brain cancers are the leading cause of cancer-related deaths in children. Biological changes in these tumors likely include epigenetic deregulation during embryonal development of the nervous system. Histone acetylation is one of the most widely investigated epigenetic processes, and histone deacetylase inhibitors (HDACis) are increasingly important candidate treatments in many cancer types. Here, we review advances in our understanding of how HDACis display antitumor effects in experimental models of specific pediatric brain tumor types, i.e., medulloblastoma (MB), ependymoma (EPN), pediatric high-grade gliomas (HGGs), and rhabdoid and atypical teratoid/rhabdoid tumors (ATRTs). We also discuss clinical perspectives for the use of HDACis in the treatment of pediatric brain tumors.

## Introduction

Brain tumors of the childhood represent the leading cause of cancer-related deaths in children aged 0–14 years, and survivors often present long-term neurological sequelae that impair their quality of life (Ostrom et al., 2016). These cancer types include medulloblastoma (MB), which is the most common and most studied type of childhood brain tumor, ependymoma (EPN), pediatric high-grade gliomas (HGGs), and rhabdoid and atypical teratoid/rhabdoid tumors (ATRTs) (Guerreiro Stucklin et al., 2018). Pediatric brain cancers may originate from defects in embryonal development affecting cell types including neural stem cells (NSCs) and neuronal precursors, or dedifferentiation of mature neuronal or glial cells (Taylor et al., 2005; Visvader, 2011; Liu and Zong, 2012; Wang and Wechsler-Reya, 2014; Azzarelli et al., 2018).

Normal development, cellular differentiation, and tissue specialization are finely regulated by various epigenetic mechanisms (Atlasi and Stunnenberg, 2017). Epigenetic regulation allows changes in chromatin structure that control gene expression without modifications in DNA sequence. Histone acetylation and DNA methylation feature among the most widely investigated epigenetic mechanisms (Surani et al., 2007). Histone acetyltransferases (HATs) and deacetylases (HDACs) play opposing roles in regulating gene expression. HATs transfer acetyl groups to the amino-terminal lysine residues of histones, thus increasing histone acetylation and transcriptional activity. On the other hand, HDACs remove acetyl groups, thus promoting chromatin condensation and overall repression of gene expression, in addition to displaying other, non-epigenetic actions (Kouzarides, 2007; Sanaei and Kavoosi, 2019; D’Mello, 2020; Milazzo et al., 2020). Changes in the expression and activity of HATs and HDACs were described in leukemias, and afterward in solid tumors, and in many cases decreased levels of histone acetylation were shown to correlate with clinical outcome. Therefore, HDAC inhibitors (HDACis) have become promising and widely investigated experimental anticancer agents (Lane and Chabner, 2009; Li and Seto, 2016).

Pediatric cancers hijack and modify biological processes involved in normal embryonic development, including epigenetic modifications, to promote tumor progression (Liu and Zong, 2012; Marshall et al., 2014). An important component of childhood cancer biology is epigenetic reprogramming that can lock cells in a stem cell-like, poorly differentiated and highly proliferative phenotype (Lawlor and Thiele, 2012). Accumulating evidence implicates epigenetic abnormalities in the genesis and progression of pediatric brain tumors. The relationship between epigenetic markers and patient survival has been investigated (Bhattacharya et al., 2020), and epigenetic modulators have shown promising effects in experimental models. Here we review the potential of HDACis in the treatment of selected types of brain cancer that afflict children.

## Epigenetic Basis of Pediatric Brain Cancers

Mutations and genetic variations affecting epigenetic-regulating mechanisms are features of several types of childhood brain cancers, including MB, EPN, ATRT, and pediatric gliomas (Mack and Taylor, 2009; Parsons et al., 2011; Dubuc et al., 2012; Jones et al., 2012; Lee et al., 2012; Northcott et al., 2012; Buczkowicz et al., 2014; Mack et al., 2016). In fact, MB and low-grade gliomas are among the types of pediatric tumors with highest frequency of mutation in genes encoding epigenetic regulators (Huether et al., 2014). MicroRNAs (miRNAs) regulate pediatric brain tumor cells at posttranscriptional/translational levels, acting on a range of functional aspects linked to cancer progression, including proliferation and stemness. The diagnostic, prognostic, and therapeutic potential of miRNAs has been increasingly highlighted (Garg et al., 2015; Leichter et al., 2017; Pezuk et al., 2019). Epigenetic remodeling genes SMARCB1 and SMARCA4 are among the most frequently altered in pediatric brain tumors, with most cases of ATRT showing changes in those genes (Frühwald et al., 2016; Wang et al., 2017; Johann, 2020). Enhancer of zeste homolog 2 (EZH2), which is part of a Polycomb repressor complex that methylates lysine 27 of histone H3 (H3K27), leading to transcription inhibition, is often mutated or highly expressed in pediatric brain tumors (Huether et al., 2014; Li et al., 2015; Kim and Roberts, 2016; Erkek et al., 2019; Zhang et al., 2020).

A reduced histone acetylation state can contribute to cancer through repressing differentiation and tumor suppressor genes while allowing overexpression of genes promoting proliferation. Chromatin modifications mediated by histone acetylation can also epigenetically influence the tumor genetic landscape, for example by leading to DNA copy gain in the absence of chromosomal instability (Black et al., 2013; Mack et al., 2016). HDACs play a central role in epigenetic regulation through reducing acetylation. It is currently known that some HDACs can either repress or activate gene transcription, in addition to displaying non-epigenetic activities by acting directly on nuclear and cytoplasmic proteins (D’Mello, 2020). For example, transcription factors E2F1, STAT1, STAT3, and NF-κB can be directly hyperacetylated by HDACis (Johnstone and Licht, 2003; Glozak et al., 2005; Bolden et al., 2006).

Histone acetyltransferases and deacetylases are currently classified into different classes. Class I HDACs include nuclear HDAC1, 2, 3, and 8. Class II HDACs occur in both the nucleus and cytoplasm and are classified into two subclasses: HDAC4, 5, 7, and 9 are grouped as IIa, whereas HDAC6 and 10 are classified as class IIb. Sirtuins constitute Class III HDACs, being found in the nucleus, cytoplasm, and mitochondria according to the specific type. Finally, HDAC11 has been proposed as a Class IV HDAC (Falkenberg and Johnstone, 2014; Hassell, 2019; D’Mello, 2020; Milazzo et al., 2020). Most deacetylase activity in mammalian cells has been attributed to Class I HDACs (Lahm et al., 2007).

Increased HDAC2 expression (Park et al., 2003), deletions or amplifications of histone methyltransferases and demethylases (Northcott et al., 2009), DNA hypermethylation (Frühwald et al., 2001), and altered miRNA expression (Ferretti et al., 2008) have been reported in MB. High HDAC5 and HDAC9 expression is found in prognostically poor MB subgroups and significantly associated with poor overall survival, posing an independent risk factor (Milde et al., 2010). Other alterations in epigenetic components found in MB include truncating mutations in the KDM6A gene encoding a histone 3 lysine 27 demethylase (Jones et al., 2012), homozygous deletions of genes involved in histone lysine methylation, and amplification of the HAT gene MYST3 (Northcott et al., 2009). Expression of HDAC2 is higher in patients with MB subgroups with poor prognosis (sonic hedgehog (SHH), Group 3 and Group 4), and *MYC*-amplified MB cell lines show increased mRNA levels of class I HDACs compared to the normal cerebellum (Ecker et al., 2015).

Hypermethylation of the transcriptional repressor hypermethylated in cancer 1 (*H1C-1*) was identified in 83% of EPN samples (Waha et al., 2004). Global reduction of H3K27me3 analogous to H3K27M mutant gliomas, accompanied by CpGi hypermethylation, is found in EPN (Bayliss et al., 2016). Poor-prognosis EPNs show a CpG island methylator phenotype, where transcriptional silencing driven by CpG methylation converges on targets of the Polycomb repressive complex 2 (PRC2), which represses expression of differentiation genes through trimethylation of H3K27 (Mack et al., 2014). Posterior fossa type A (PFA) EPNs show low H3K27 methylation and high levels of Enhancer of Zeste Homologs Inhibitory Protein (*EZHIP*), which promotes a similar chromatin state compared to H3K27M (Jain et al., 2019). Enrichment of 5-carboxylcytosine (5caC) and increased *TET1* expression, which are involved in active DNA demethylation, are epigenetic hallmarks of EPN and SHH MB (Ramsawhook et al., 2017). Hypermethylated genes in EPN converge on defined sets of embryonic stem cell (ESC) targets, suggesting a linkage, mediated by epigenetic programming, between embryonic development and pediatric brain cancer (Sin-Chan and Huang, 2014; Mack et al., 2016).

Somatic mutations in the H3.3-ATRX-DAXX chromatin remodeling pathway and recurrent mutations in the gene encoding the histone 3 variant H3.3 are highly prevalent in pediatric glioblastoma (Schwartzentruber et al., 2012). In diffuse intrinsic pontine glioma (DIPG), a deadly type of childhood glioblastoma, a mutation that leads to hypomethylation by replacing a lysine to methionine (K27M) on H3F3A and HIST1H3B/C genes encoding histone variants is the most frequent mutation (Wu et al., 2012, 2014; Mendez et al., 2020). Supporting the link between embryonic development and the arising of pediatric brain tumors, this histone mutation can contribute to resetting neural progenitors derived from human ESCs to a stem cell state, ultimately resulting in neoplastic transformation (Funato et al., 2014).

In ATRTs, HDAC1 is significantly differentially expressed (Sredni et al., 2013), and the chromatin remodeling and tumor suppressor gene SMARCB1 represses Cyclin D1 transcription by recruiting the HDAC1 complex to its promoter, resulting in cell cycle arrest (Tsikitis et al., 2005). A hallmark of malignant rhabdoid tumors is homozygous deletion or inactivation of SMARCB1. Histone acetylation and methylation patterns, as well as HDAC and HAT levels, are influenced by insulin-like growth factor receptor 1 (IGF-1R) signaling (Shim et al., 2013). For comprehensive reviews on the role of epigenetic changes as part of the biological basis of pediatric brain cancers, see Dubuc et al. (2012) and Mack et al. (2016).

## Effects of HDAC Inhibition in Experimental Pediatric Brain Cancers

Most HDACis widely used experimentally or clinically preferentially inhibit Class I and II HDACs. These agents include sodium butyrate (NaB), trichostatin A (TSA), valproic acid (VPA), suberoyl anilide hydroxamic acid (SAHA, vorinostat), panobinostat, belinostat, and romidepsin (Bolden et al., 2006; Li and Seto, 2016; Millard et al., 2017; Hassell, 2019). HDACis induce anticancer effects in several experimental tumor types by targeting aberrant chromatin alterations, resulting in changes in cell proliferation, viability, differentiation, migration, and angiogenesis (Bolden et al., 2006; Sanaei and Kavoosi, 2019; Ribatti and Tamma, 2020). In addition to modulating acetylation by inhibiting HDACs, HDACis may directly modulate miRNAs and also alter protein kinase signaling through acetylation-independent mechanisms (Chen et al., 2005; Autin et al., 2019). The HDACi TSA inhibits HDAC6, a predominantly cytoplasmic HDAC, which likely induces many effects independent of alterations in gene expression stimulated by histone acetylation (Johnstone and Licht, 2003; Chen et al., 2005; Glozak et al., 2005). When combined with agents targeting other epigenetic regulators, such as EZH2, HDACis modulate acetylation and methylation of H3K27, through mechanisms involving PRC2 complex disruption (Lue et al., 2019). Below, we summarize studies examining the effects of HDACis in experimental models of pediatric brain tumors.

### Medulloblastoma

Medulloblastoma is currently classified within four distinct molecular subgroups, namely, WNT, SHH, Group 3, and Group 4, with subtypes within each group being now recognized (Louis et al., 2016). An early study by Jaboin et al. (2002) showed that the HDACi MS-275 inhibits proliferation of Daoy and D283 Med MB cells. A subsequent study by Li and colleagues showed that VPA, which partially acts as a class I and II HDACi, when used at clinically safe concentrations, leads to growth inhibition, cell cycle arrest, apoptosis, senescence, differentiation, and inhibition of colony formation in Daoy and D283 Med cells. In addition, daily systemic injection of VPA (400 mg/kg) for 28 days significantly inhibits *in vivo* growth of Daoy and D283 Med xenografts in immunodeficient mice. These effects are associated with hyperacetylation of histone H3 and H4, activation of p21, and suppression of *TP53*, *CDK4*, and *c-MYC* (Li et al., 2005). The HDACis SAHA, NaB, and TSA induce apoptotic cell death related to dissipation of mitochondrial membrane potential and activation of caspase-9 and -3 in Daoy and UW228-2 MB cells. These HDACis also enhance the cytotoxic effects of ionizing radiation in Daoy cells, and treatment with SAHA potentiates the cytotoxic actions of etoposide and tumor necrosis factor-related apoptosis-inducing ligand (TRAIL), but not vincristine (Sonnemann et al., 2006). HDACi-induced TRAIL sensitization is associated with increased caspase-8 activation (Sonnemann et al., 2012). VPA combined with interferon (IFN)-gamma restores caspase-8 expression and sensitivity to TRAIL in primary MB samples and significantly potentiates TRAIL-mediated suppression of MB growth *in vivo* (Häcker et al., 2009). HDACi potentiation of ionizing radiation effects in MB cells was also reported by Kumar et al. (2007). A variety of HDACis, including MS-275, SAHA, TSA, and VPA, are able to inhibit proliferation of MB cell lines and induce histone H4 hyperacetylation, reactivation of expression of growth regulatory genes, and induction of apoptosis, as well as reduction of MB xenograft growth *in vivo* (Furchert et al., 2007). HDACis helminthosporium carbonum (HC)-toxin, SAHA, and panobinostat reduce viability and lead to radiosensitization accompanied by increased cell death in the HD-MB03 cell line, a preclinical model of Group 3 MB (Milde et al., 2012). Inhibition of class I HDACs in MB cells reduces metabolic activity, cell number, and viability and enhances sensitivity to HDACi specifically in *MYC*-amplified cells (Ecker et al., 2015). Histone-mediated deregulation of expression of the Wnt antagonist Dickkopf-1 (DKK1) impairs its tumor-suppressing activity and contributes to experimental MB tumorigenesis, and treatment with TSA restores DKK1 in D283 Med cells (Vibhakar et al., 2007). TSA significantly inhibits telomerase activity, increases expression of p53 and p21, and reduces cyclin-D levels in ONS-76 MB cells. Upregulation of Bax and cytochrome c correlates with pro-apoptotic effects in TSA-treated cells (Khaw et al., 2007).

HDAC-mediated deacetylation of Gli (glioma-associated oncogene) promotes transcriptional activation through Hedgehog (Hh)-induced upregulation of HDAC1, and loss of HDAC activity hinders Hh pathway-dependent growth of neural progenitors and MB cells (Canettieri et al., 2010; De Smaele et al., 2011). The functional interaction between the transcription cofactor ZNF521 and GLI1 and GLI2, which enhances Hh signaling, is sensitive to HDACis (Scicchitano et al., 2019). Hh signaling stimulates granule precursor (CGP) proliferation during the early stages of postnatal cerebellar development and sustains HDAC activation leading to stimulation of CGPs. HDAC inhibition impairs Shh-induced CGP proliferation and improves aberrant CGP proliferation in a mouse model of MB (Lee et al., 2013). NL-103, a dual-targeted inhibitor of both HDAC and Hh signaling, effectively overcame resistance to the Hh inhibitor vismodegib (Zhao et al., 2014). HDAC6 is an important regulator of the Hh pathway, and selective HDAC6 inhibition hinders MB cell survival *in vitro* and reduces tumor growth in an *in vivo* allograft model (Dhanyamraju et al., 2015). HDACis show more pronounced effects on proliferation of SHH-driven MB cells harboring a mutation in the gene encoding for the histone acetyltransferase (HAT) CREBBP, when compared to CREBBP wild-type controls (Hellwig et al., 2019). A targeted small-molecule screen on the stable, SHH-dependent murine MB cell line SMB21 reveals selective inhibitors of class I HDACs as promising antitumor agents for SHH MB, and the novel class I HDAC inhibitor JNJ-26481585 (quisinostat) consistently inhibits growth of SHH MB *in vivo* as well as *in vitro* (Pak et al., 2019). Another recent study using a high-throughput cell viability assay to screen 12,800 compounds identified two HDACis, JNJ-26481585 and dacinostat, as anti-proliferative agents in MB. Both compounds induce cytotoxicity and apoptosis and block cell cycle progression at the G2/M phase, in addition to reducing the growth of MB xenografts in mice (Zhang et al., 2019).

Histone deacetylase inhibitors sensitize MB cells to apoptosis induced by cytotoxic chemotherapy via an enhancement of p53-dependent Bax activation (Häcker et al., 2011). The HDACi sodium butyrate (NaB) at a low dose more effectively inhibits D283 cell viability when combined with the chemotherapeutic etoposide (Nör et al., 2013). SAHA combined with 13-cis retinoic acid (RA) induces apoptosis and transcription of bone morphogenetic protein-2 (BMP-2) in MB cells and is more effective than each drug alone in inhibiting MB growth *in vitro* and *in vivo*. Moreover, intracranial MB tumors in mice treated with SAHA plus RA plus cisplatin show a 4-fold increase in apoptosis over controls, and a 2-fold increase over animals receiving only SAHA or RA plus SAHA (Spiller et al., 2008). The combination of RA with epigenetic modulators in based upon functional interplays among retinoid receptors, histone acetylation, and DNA methylation. For example, HDACs bind to RA response elements in proximal promoters or enhancer regions of genes regulated by retinoids in stem cells, and retinoid receptors interact with the transcription complex mediating the placement or removal of epigenetic marks on histones and DNA (Gudas, 2013; Urvalek and Gudas, 2014; Almeida et al., 2017). Combinations of HDACis with other epigenetic modulators have also been evaluated. SAHA plus the DNA methyltransferase 1 (DNMT) inhibitor 5-aza-2′-deoxycytidine (5-aza-dC) produce a synergistic effect on survival of Daoy and D283 Med MB cells (Yuan et al., 2017).

Combining HDACis with growth factor receptor ligands such as receptor tyrosine kinase (RTK) inhibitors (gefitinib or vandetanib) or brain-derived neurotrophic factor (BDNF) is also more effective than each agent given alone in impairing MB cell viability (Marino et al., 2011; Nör et al., 2011). However, bombesin receptor antagonists failed to potentiate the effects of HDACi inhibition (Jaeger et al., 2016). Other studies have investigated combinations of HDACis with protein kinase signaling inhibitors. The multi-kinase inhibitor sorafenib and VPA interact to radiosensitize and kill MB cell lines (Tang et al., 2012). SAHA shows additive cytotoxicity with the Aurora A kinase inhibitor MLN8237 in Daoy cells (Muscal et al., 2013a). A study by Geron et al. (2015) examined the effects of the pan-aurora kinase inhibitor AMG 900 alone or in combination with SAHA in UW402, UW473, and ONS-76 MB cells. A synergistic effect of combining AMG 900 and SAHA is observed on cell proliferation in all these cell lines, especially in sequential drug treatment. The drug combination also fully inhibits cell survival measured by colony formation. Using an animal model of *MYC*-driven MB to screen for promising drugs, Pei et al. (2016) found HDACis among the most effective compounds. HDACis potently inhibit survival of *MYC*-driven MB cells, through a mechanism involving expression of the FOXO1 tumor suppressor gene. Importantly, HDACis synergize with phosphatidylinositol 3-kinase (PI3K) inhibitors to inhibit MB growth *in vivo*. NaB reduces viability and increases acetylation in human MB cells, the anti-proliferative effect of NaB being enhanced by combination with a mitogen-activated protein kinase (MAPK)/extracellular-related kinase (ERK) inhibitor (Jaeger et al., 2020).

### Ependymoma

Ependymomas featuring a CpG island methylator phenotype respond to drugs that target DNA or H3K27 methylation, revealing epigenetic modulators as the first rational therapeutic candidates in this tumor type (Mack et al., 2014). In a high-risk cytogenetic group 3 and molecular group C EPN model (DKFZ-EP1NS) that shows high tumorigenic potential *in vivo*, cells are resistant to cytotoxic chemotherapeutics temozolomide, vincristine, and cisplatin but respond to HDACi treatment (Milde et al., 2011).

### Diffuse Intrinsic Pontine Glioma

A seminal study using a chemical screen in patient-derived DIPG cultures along with RNA-seq analyses and integrated computational modeling to identify potentially effective therapeutic strategies has highlighted the potential of HDACs as targets. Importantly, the HDACi panobinostat showed antitumor efficacy both *in vitro* and in orthotopic xenograft models. Furthermore, combination of panobinostat with the histone demethylase inhibitor GSK-J4 showed that the two had synergistic effects (Grasso et al., 2015). Another study of panobinostat in experimental DIPG found that it effectively impaired cell proliferation, viability, and clonogenicity and induced apoptosis in human and murine DIPG cells. In genetically engineered tumor-bearing mice, panobinostat reduced tumor growth and increased H3 acetylation. Extended daily treatment of both genetic and orthotopic xenograft models with 10 or 20 mg/kg panobinostat led to significant toxicity, while reduced, well-tolerated doses of panobinostat failed to prolong overall survival (Hennika et al., 2017). In DIPG primary cells, panobinostat potentiated the effects of gene therapy based on human adipose tissue-derived mesenchymal stem cells expressing the secreted form of TRAIL (hAT-MSC.sTRAIL), inducing a decrease in tumor volume and prolonging survival (Choi et al., 2019).

Combined treatment with the HDACi panobinostat and the AXL inhibitor BGB324 resulted in synergistic antitumor effects on DIPG cells, with reduced expression of genes related to mesenchymal phenotype, stemness, and DNA damage repair (Meel et al., 2020). HDACis also synergize with blockade of bromodomain inhibition or CDK7, which disrupts oncogenic transcription, in DIPG models. HDAC inhibition by panobinostat, together with the bromodomain inhibitor JQ1 or the CDK7 inhibitor THZ1, synergistically reduced cell viability in DIPG cell cultures and proved more effective than single-drug treatments in inhibiting proliferation and inducing apoptosis. Panobinostat and JQ1 induced overlapping transcriptional changes, downregulating many of the same sets of genes (Nagaraja et al., 2017). VPA potentiates carboplatin cytotoxicity and increases histone H3 acetylation in different DIPG cell lines (Killick-Cole et al., 2017). CUDC-907, a first-in-class dual inhibitor of HDACs and PI3K, is a potent cytotoxic agent in DIPG models. Mechanisms underlying CUDC-907 actions include regulation of DNA damage response. It also displays radiosensitizing effects mediated by decreased nuclear factor kappa B (NFκB)/Forkhead box M1 (FOXM1) recruitment to promoters of genes involved in response to DNA damage (Pal et al., 2018). A CRISPR screen showed that knockout of KDM1A encoding lysine-specific demethylase 1 (LSD1) sensitizes DIPG cells to HDACis. Corin, an HDAC and LSD1 inhibitor, hinders *in vitro* and *in vivo* DIPG growth by increasing H3K27me3 levels as well as HDAC-targeted H3K27ac and LSD1-targeted H3K4me1 at differentiation-associated genes (Anastas et al., 2019).

### Rhabdoid and Atypical Teratoid/Rhabdoid Tumors

Atypical teratoid/rhabdoid tumors cell proliferation is impaired by a variety of HDACis, including MS-275, SAHA, TSA, and VPA (Jaboin et al., 2002; Furchert et al., 2007), and pretreatment with HDACis potentiates the effect of ionizing radiation on ATRT cells as measured by a colony-formation assay (Blattmann et al., 2012; Knipstein et al., 2012). SAHA shows synergism with doxorubicin and the cyclinD1 inhibitor fenretinide in inhibiting proliferation of rhabdoid cells (Kerl et al., 2013). Objective ATRT tumor regressions accompanied by increases in histone acetylation were observed in mice after treatment with the natural tetrapeptide HDACi depsipeptide (Graham et al., 2006). The HDACi FK228 (depsipeptide) induces autophagy in malignant ATRT cells by inducing apoptosis inducing factor (AIF) translocation to the nucleus (Watanabe et al., 2009). The cell cycle inhibitor CDKN1C, a tumor suppressor in ATRT, is activated by HDACis (Algar et al., 2009). SAHA combined with fractionated irradiation significantly reduces tumor growth in rhabdoid xenografts (Thiemann et al., 2012). A recent study showed that the epigenetic modulating compound domatinostat (4SC-202), which inhibits both class I HDACs and lysine demethylase (LSD1), displays cytotoxic and cytostatic actions in 2D and 3D ATRT scaffold cell culture models and reduces expression of stemness genes (Hoffman et al., 2020).

## The Role of Modulating Stemness and Differentiation

As discussed above, abnormal epigenetic programming in pediatric cancers may lock tumor cells in a stem cell-like, poorly differentiated state (Lawlor and Thiele, 2012; Marshall et al., 2014). Pediatric brain tumors often display upregulation of genes known as markers of neural stem cells, including *CD133*, *Nestin*, and *Musashi*, and deregulation of other genes that regulate stemness is frequently found (Bahmad and Poppiti, 2020).

HDACis may act partially by restoring expression of prodifferentiation genes, thus influencing tumor cell phenotype toward a less malignant state. In fact, NaB increases the mRNA expression of the neuronal differentiation marker Gria2 in D283 and Daoy cells (Nör et al., 2013). The HDACi panobinostat suppresses leptomeningeal seeding (a strong negative prognostic factor) and prolongs survival in an animal model of MB, while also inducing formation of neurophil-like processes and promoting expression of synaptophysin and NeuroD1, suggesting neuronal differentiation (Phi et al., 2017). Corin promoted change to a more differentiated phenotype in experimental DIPG (Anastas et al., 2019). Treatment with low doses of HDACis can induce terminal differentiation of rhabdoid cells and reduce their ability to self-renew (Muscat et al., 2016).

Given the proposed role of cancer stem cells (CSCs) in the progression, recurrence, and metastasis of brain cancers, studies have also investigated whether HDACis can reduce stemness in MB. Treatment with NaB reduces the formation of MB neurospheres, a model of enriching putative cancer stem cells in culture (Nör et al., 2013). Analyses of MB tumor samples from patients reveals expression of the stemness markers *BMI1* and *CD133* in all MB molecular subgroups, and NaB is able to reduce *BMI1* and *CD133* expression in cultured MB cells (Jaeger et al., 2020). In the DKFZ-EP1NS model of EPN, SAHA induces neuronal differentiation associated with loss of stemness (Milde et al., 2011). As noted above, HDACi effects in H3K27M DIPG cells involve a decrease in stemness gene expression (Meel et al., 2020). These findings support the view that HDACis should be further investigated as prodifferentiating and stemness modulating agents in pediatric brain tumors. A summary of studies examining the effects of pharmacological inhibition of HDACs in the tumor types reviewed here is presented in Table 1.

**TABLE 1:** Research findings from selected experimental studies examining the effects of HDACis in experimental models of pediatric brain tumors.

**Tumor type**	**Model**	**HDACis used**	**Main findings**	**References**
MB	Daoy and D283 Med, *in vitro*	MS-275	Reduced cell proliferation	Jaboin et al., 2002
MB	Daoy and D283 Med, *in vitro*	VPA	Reduced cell growth and survival, cell cycle arrest; induction of apoptosis, senescence, and neuronal and glial differentiation; hyperacetylation of histones H3 and H4, activation of p21, and suppression of *TP53*, *CDK4*, and *c-MYC*	Li et al., 2005
MB	Daoy and D283 Med, subcutaneous (s.c.) xenografts in severe combined immunodeficient mice	VPA, daily systemic injections for 28 days	Reduced tumor growth *in vivo*	Li et al., 2005
MB	Daoy and UW228-2, *in vitro*	SAHA, NaB, and TSA	Impairment of mitochondrial membrane potential, activation of caspase-9, -8, and -3, apoptotic cell death; enhancement of cytotoxicity induced by ionizing radiation (IR), etoposide, and TRAIL	Sonnemann et al., 2006, 2012
MB	Daoy and UW228-2, *in vitro*	SAHA and TSA	Enhancement of IR-induced cytotoxicity and cell cycle arrest	Kumar et al., 2007
MB	D458 and primary MB cultures *in vitro*, chorioallantoic membrane model using D458 cells	MS-275, VPA, and SAHA	Cooperation with cytotoxic chemotherapeutics to induce loss of mitochondrial membrane potential, cytochrome c release, and caspase-dependent apoptosis and reduce cell survival and tumor growth	Häcker et al., 2009
MB	D341 Med, Daoy, MHH−PNET−5, and UW228−2, *in vitro*	MS−275, SAHA, TSA, M344, M360, D85, SW55, SW187, and VPA	Reduced cell proliferation, hyperacetylation of H4, reactivation of genes including CASP8; induction of apoptosis	Furchert et al., 2007
MB	HD-MB03, *in vitro*	Helminthosporium carbonum (HC)-toxin, SAHA, and panobinostat	Induction of cell death, sensitization to radiation-induced cell death	Milde et al., 2012
MB	MED8A, UW228-2, ONS76, Daoy, HD-MB03, and D458, *in vitro*	siRNA-mediated knockdown of HDAC2; HDACis MAZ1863, MAZ1866, SAHA, and MS-275	Reduced metabolic activity, cell number, and viability; increased sensitivity to HDACi in *MYC*-amplified cells; increased H4 acetylation and cell death after HDAC2 knockdown; *in vitro* simulation of clinical pharmacokinetics showed time-dependent on-target activity correlated with binding kinetics of HDACis	Ecker et al., 2015
MB	D283 Med, *in vitro*	TSA	Upregulation of Dickkopf-1 (DKK1), a Wnt antagonist	Vibhakar et al., 2007
MB	ONS-76, *in vitro*	TSA	Inhibition of telomerase activity, increased expression of p53 and p21, and reduced cyclin-D levels; upregulation of Bax and cytochrome c correlates with TSA-induced apoptosis	Khaw et al., 2007
MB	HEK293T, *in vitro*	TSA, NaB, and VPA	Inhibition of ZNF521 cooperation with GLI1 and GLI2 in the transcriptional activation of GLI-responsive promoters	Scicchitano et al., 2019
MB	Primary culture of CGP cells from Smo/Smo mice, *in vitro*	TSA and tubastatin	Impairment of Shh-induced CGP proliferation and improvement of aberrant CGP proliferation	Lee et al., 2013
MB	NIH3T3−12Gli, *in vitro*	NL-103, SAHA	Decreased resistance to vismodegib, inhibition of the Shh pathway	Zhao et al., 2014
MB	NIH3T3, Hek293A, ShhL2, and C3H10T1/2, *in vitro*	TSA, ACY-1215, CAY-10603, tubacin	Inhibition of Hh signaling and expression of genes encoding for components of the Hh pathway	Dhanyamraju et al., 2015
MB	S.c. injection of primary MB99-1 cells from SmoA1 MB E1270) into C57BL/6J mice	S.c. administration of ACY-1215 (50 mg/kg on days 0, 1, 2, 3, 4, 7, 8, 9, 10, and 11 after tumors were palpable	Reduced tumor growth	Dhanyamraju et al., 2015
MB	Cre-dependent *in vitro* system for SHH-driven MB based on cultured primary cerebellar granule neuron precursors from *SmoM2*^Fl/+^ and *Crebbp^Fl/F^lSm oM2^Fl/+^* mice	TSA	Preferential reduction in cell proliferation and tumor growth in SHH-driven MB harboring a CREBBP mutation	Hellwig et al., 2019
MB	*Math1-creER^T2^::SmoM2^Fl/+^* mice with CREBBP-mutated SHH MB	Intraperitoneal (i.p.) administration of TSA (0.5 μg/g, once daily) or LBH-589 (panobinostat, 20 μg/g, once weekly) for 30 days	Preferential reduction of tumor growth in SHH-driven MB harboring a CREBBP-mutation	Hellwig et al., 2019
MB	SMB21 (SHH-dependent murine MB cell line) and its mutant derivatives, *in vitro*	JNJ-26481585 (quisinostat), DLS-3, MERCK 60, WT 161, OJI-1, and pandacostat	Inhibitor-specific reduction of cell viability, including SMO-resistant SHH cells, and inhibition of the SHH pathway	Pak et al., 2019
MB	S.c. xenografts into the flank of nude mice; and endogenously arising intracranial SHH MB model in *Atoh1-cre::SmoM2^Fl/+^* mice	Systemic administration of JNJ-26481585 (8 mg/kg) daily, starting at postnatal day P20	Survival benefit, reduction in expression of SHH target genes *Gli1* and *Ptch1*, good pharmacokinetic profile	Pak et al., 2019
MB	Daoy and D283 Med, *in vitro*	JNJ-26481585 or dacinostat	Reduced cell viability, apoptosis, and Akt phosphorylation; G2/M cell cycle arrest; increased histone H3 and H4 acetylation	Zhang et al., 2019
MB	S.c. Daoy xenografts in the flank of NSG mice	I.p. administration of JNJ-26481585 or dacinostat (20 mg/kg) every 2 days starting when tumor volumes reached 100 mm^3^	Inhibition of tumor growth and cell proliferation and increased apoptosis	Zhang et al., 2019
MB	D458 and primary cultures, *in vitro*	MS-275, VPA, SAHA	Increased histone H3 and Ku70 acetylation; MS-275 pretreatment-induced enhancement of apoptosis triggered by doxorubicin, etoposide, cisplatin, and topotecan	Häcker et al., 2011
MB	Chorioallantoic membrane model using D458 cells	MS-275	Increased efficacy in inhibiting tumor growth when combined with doxorubicin	Häcker et al., 2011
MB	Daoy and D283 Med, *in vitro*	NaB	Increased cell death and expression of the neuronal marker Gria2; reduced neurosphere formation; potentiation of cytotoxic effect of etoposide	Nör et al., 2013
MB	D283 Med, *in vitro*	SAHA	Enhancement of RA-mediated BMP-2 transcription; induction of apoptosis-mediated cell death potentiated by combination with RA	Spiller et al., 2008
MB	s.c. D283 xenografts injected into the flank of nude mice; intracranial MB in ND2:SmoA1 transgenic mice	SAHA mixed into powdered food at a final concentration of 200 mg/kg/day for up to 21 days (nude mice); i.p. injections of SAHA (200 mg/kg) daily for 3 days in ND2:SmoA1 transgenic mice	Reduced tumor growth with oral SAHA combined with RA in nude mice; increased apoptosis in intracranial tumors and lack of dose-limiting hematopoietic toxicity in ND2:SmoA1 mice	Spiller et al., 2008
MB	Daoy and D283 Med, *in vitro*	TSA, SAHA, parthenolide, mocetinostat, tacedinaline, romidepsin	Reduced oxygen-dependent cell viability, induction of apoptosis, reduced expression of the stem cell marker CD133, reduced tumorsphere survival. Increased cytotoxic and proapoptotic effects when combined with the DNMT inhibitor 5-aza-dC	Yuan et al., 2017
MB	Daoy, *in vitro*	4-Phenylbutyrate	Reduced cell viability only when combined with gefitinib; potentiation of effects of gefitinib and vandetanib on cell survival	Marino et al., 2011
MB	Daoy and ONS76, *in vitro*	NaB	Reduction in cell viability by HDACis combined with human recombinant BDNF	Nör et al., 2011
MB	D283 Med, *in vitro*	NaB	Reduction of cell viability by NaB alone or combined with bombesin receptor antagonists	Jaeger et al., 2016
MB	Daoy and D283 Med, *in vitro*	VPA, SAHA	Induction of cell death through activation of the extrinsic pathway when combined with sorafenib	Tang et al., 2012
MB	Daoy, *in vitro*	SAHA	Increased histone acetylation, induction of cytotoxicity, synergistic effects when combined with MLN8237	Muscal et al., 2013a
MB	UW402, UW473, and ONS-76, *in vitro*	SAHA	Reduced cell proliferation and survival alone or combined with AMG 900	Geron et al., 2015
MB	Patient-derived lines including MB002, ICb-984, ICb-1572, ICb-1487, ICb-1299, Med-1712-FH, Med-411-FH, Med-211-FH, RCMB28, RCMB18, RCMB32, DMB006	SAHA, HNHA, LBH-589, Scriptaid, MS-275, givinostat, PDX 101, LAQ-824, and MGCD0103	Inhibition of cell survival through a mechanism targeting FOXO1; synergistic effect with PI3K inhibitors	Pei et al., 2016
MB	*In vivo* intracranial tumors generated in NOD-SCID IL2R-gamma null (NSG) mice, from cerebellar stem/progenitor cells (Prom1+ cells) infected with Myc-IRES-Luciferase and DNp53-IRES-GFP retroviruses and stereotaxically injected into the cerebellum of 6- to 8-week-old mice	LBH-589 (5 mg/kg) given i.p., in 4-day cycles (3 days on, 1 day off)	Reduced phosphorylation of Akt and S6, increased histone acetylation and FOXO1 content, reduced tumor growth, and prolonged survival; cooperation with the PI3K inhibitor BKM-120	Pei et al., 2016
MB	Daoy and D283 Med, *in vitro*	NaB	Reductions in cell viability and expression of *BMI1* and *CD133*; increased acetylation; anti-proliferative effect potentiated by combination with the MAPK/ERK inhibitor U0126	Jaeger et al., 2020
MB	UW228, UW426, MED8A, *in vitro*	Panobinostat	Reduced cell viability, migration, and adhesion, cell cycle arrest, induction of apoptosis, and neuronal differentiation	Phi et al., 2017
MB	Orthotopic injection of UW426-effLuc cells in nude mice	Systemic administration of panobinostat (10 mg/kg) every 5 days for 2 weeks	Reduction of tumor growth and leptomeningeal seeding, prolonged survival	Phi et al., 2017
MB	Daoy and D283 Med, *in vitro*	FTY720	Reduced cell viability and survival, increased H3 acetylation	Perla et al., 2020
EPN	DKFZ-EP1NS, *in vitro*	SAHA, panobinostat, MS-275, and VPA	Reduced metabolic activity and neurosphere formation capability; induction of neuronal differentiation; loss of stemness; G0–G1 cell cycle arrest	Milde et al., 2011
DIPG	SU, NEM, JHH. Li, VU, JHH-DIPG-1, SF7761, and primary cultures, *in vitro*	Panobinostat and SAHA	Decreased cell viability, increased H3 acetylation and H3K27-trimethylation, normalization of K27M gene expression signature, and decrease in *MYC* gene signature; synergism with GSK-J4	Grasso et al., 2015
DIPG	DIPG orthotopic xenograft mouse model, using cells from SU-DIPG-VI-luc neurospheres and NOD-SCID-IL2 gamma chain-deficient mice	Pabinostat infused into the pons or given via i.p. injections	Prolonged survival	Grasso et al., 2015
DIPG	HSJD-DIPG-007	Panobinostat	Reduced cell survival	Hennika et al., 2017
DIPG	Autochthonous PDGF-B;H3.3-K27M;p53-deficient BSG genetically engineered mice and DIPG orthotopic xenograft mouse model	i.p. administration of panobinostat (10 to 20 mg/kg), once daily for three to five days	Drug delivery to the brain after systemic administration, reduced tumor cell proliferation, increased H3 acetylation	Hennika et al., 2017
DIPG	DIPG XIII: H3.3 K27M and DIPG XIX: H3.3 K27M primary cells; *in vitro*	Panobinostat	Reduced cell viability; increased expression of death receptors 4 and 5; cytotoxicity potentiated by combination with hAT-MSC.sTRAIL	Choi et al., 2019
DIPG	VUMC-DIPG-A, VUMC-DIPG-08, VUMC-DIPG-10, SU-DIPG-IV, SU-DIPG-XIII, SU-DIPG-XXI, SU-pcGBM-2, HSJD-DIPG-07, HSJD-DIPG-08, HSJD-DIPG-12, JHH DIPG-01, SF7761, SF862826, and mouse cell lines from murine primary tumors, *in vitro*	Panobinostat	Reduced cell viability and migration; reversal of mesenchymal transition; sensitization to radiation; synergism with the AXL inhibitor BGB324	Meel et al., 2020
DIPG	Orthotopic xenografts of HSJD-DIPG-07 Fluc cells in nude mice	Panobinostat (10 mg/kg/day) for four days, or on days 1–5 and 11–13; or a single administration via convection-enhanced delivery (CED, 2 μM)	Prolonged survival when combined with BGB324	Meel et al., 2020
DIPG	SU-DIPG-IV: H3.1-K27M, SU-DIPG-VI/XIII-P, SU-DIPG-XVII, SU-DIPG-XXV JHH-DIPG1, SF7761: H3.3-K27M, SU-DIPG-XIII-FL, and VUMC-DIPG-10, *in vitro*	Panobinostat	Synergistic effects with the bromodomain inhibitor JQ1 and the CDK7 inhibitor THZ1 in reducing cell viability and proliferation and inducing apoptosis; changes in expression of genes related to central nervous system development and synaptic organization and structure	Nagaraja et al., 2017
DIPG	SF7761, SF8628, and DUB-D003, *in vitro*	VPA	Reduced cell survival, increased histone H3 acetylation and apoptosis, potentiation of carboplatin cytotoxicity	Killick-Cole et al., 2017
DIPG	BT869, SF8628, and SF10693	CUDC-907	Cytotoxicity with synergism with radiotherapy for CUDC-907; cell cycle arrest, increased DNA damage and reduced DNA repair; inhibition of NFκB and FOXM1 expression and transcriptional activity	Pal et al., 2018
DIPG	Orthotopic injection of SF8628 cells in nude mice	CUDC-907 (100 mg/kg), orally for 10 days	Increased survival with CUDC-907, with potentiation when combined with radiotherapy	Pal et al., 2018
DIPG	SU-DIPGVI, SU-DIPGXIII, BT869, BT245, and HSJD-DIPG007	Panobinostat, entinostat, and Corin	Reduced cell viability, global changes in histone and chromatin modifications and gene expression, cell cycle arrest, induction of differentiation	Anastas et al., 2019
DIPG	HSJD-DIPG007 and SU-DIPGXIIIP* orthotopic xenografts in nude mice	Corin (0.03 mg) given via CED	Reduced tumor growth, increased H3K27ac and H3K27me3	Anastas et al., 2019
ATRT	G401, *in vitro*	MS-275	Reduced DNA synthesis and viability, increase in G1, induction of p21	Jaboin et al., 2002
ATRT	BT 12, BT 16, 7.92, and G401, *in vitro*	MS-275, SAHA, TSA, VPA, M344, M360, D85, SW55, SW187	Reduced cell viability, increased H3 and H4 acetylation, apoptosis induction	Furchert et al., 2007
ATRT	KHOS-2405 and A-204, *in vitro*	SAHA	Reduced cell viability, increased γH2AX expression	Blattmann et al., 2012
ATRT	BT-12, BT-16, and UPN737	SAHA, TSA, and SNDX-275	Reduced proliferation, increased p21^Waf1/Cip1^ content, induction of apoptosis, potentiation of radiation-induced inhibitor effects on cell survival	Knipstein et al., 2012
ATRT	BT-12, BT-16, G401, and A-204	SAHA, TSA, and M344	Induction of apoptosis, G2 arrest, expression of RB-, MYC-, and pluripotency-associated genes, synergistic cell growth inhibition and apoptosis induction when combined with fenretinide or chemotherapy	Kerl et al., 2013
ATRT	S.c xenografts of primary tumors in *scid* mice	Depsipeptide (4.4 mg/kg) given intravenously (i.v.) every 7 days × 3 with a second cycle of treatment starting on day 21	Reduced tumor growth, increased histone acetylation, p21 and p53 induction, cleavage of PARP	Graham et al., 2006
ATRT	G401, STM91-01, SJSC, and BT-16	Romidepsin	Increased CDKN1C mRNA expression and histone acetylation at the CDKN1C promoter, changes in allelic expression of *CDKN1C*	Algar et al., 2009
ATRT	A-204 s.c. xenografts in nude mice	SAHA (100 mg/kg) injected i.p. once daily for 8 consecutive days or for 15 days within three weeks	Reduced tumor growth with reduced cell proliferation and increased apoptosis with drug treatment alone or combined with radiotherapy	Thiemann et al., 2012
ATRT	CHLA-06-ATRT and CHLA-05-ATRT, *in vitro* and spheroid and 3D Scaffold models	4SC-202	Cytotoxicity, reduced stem cell marker expression, changes in gene networks	Hoffman et al., 2020
ATRT	G401, SJSC, STM91-01 *in vitro*	LBH589	Reduced cell viability and self-renewal, induction of senescence, increased H3 and H4 acetylation	Muscat et al., 2016
ATRT	S.c. xenografts of G401 or SJSC in nude mice	Daily i.p. injections of LBH589 (5 mg/kg)	Reduced tumor growth, increased differentiation	Muscat et al., 2016

*See main text for abbreviation definitions and further details.*

## Clinical Trials of HDACis in Pediatric Brain Tumors

A phase-I/II dose-escalation clinical trial of SAHA in pediatric patients with recurrent solid tumors including brain tumors has been reported (Witt et al., 2012). Another phase I trial and pharmacokinetic study of SAHA in children with solid tumors indicated that SAHA was well-tolerated at a dose of 230 mg/m(2)/d, with a small dose reduction required when SAHA was combined with RA (Fouladi et al., 2010). Another phase-I consortium clinical study recommended a dose and schedule of vorinostat at 230 mg/m(2)/day PO on days 1–5 and 8–12 in combination with the proteasome inhibitor bortezomib at 1.3 mg/m(2)/day i.v. on days 1, 4, 8, and 11 of a 21-day cycle, for future phase 2 studies in children with recurrent or refractory solid tumors (Muscal et al., 2013b). SAHA and a range of other epigenetic therapies have been evaluated in clinical trials of patients with DIPG (Hashizume, 2017). An ongoing multicenter, multiarm phase II and III trial investigates the effects of conventional chemotherapy with or without combination with an HDACi in patients with EPN (Merchant, 2017).

## Conclusion

Some HDACis have already been approved by the United States Food and Drug Administration (FDA) for the treatment of other cancer types (i.e., SAHA and romidepsin for the treatment of cutaneous T-cell lymphoma and belinostat and panobinostat for the treatment of peripheral T-cell lymphoma and multiple myeloma, respectively). Given the increasingly promising role of drug repurposing or repositioning in the identification of potential novel therapeutic strategies for pediatric brain tumors (Bahmad et al., 2020), those agents could be tested in clinical trials of patients with these cancer types. VPA is well tolerated in patients with childhood brain cancers, including heavily pretreated pediatric patients with HGG or DIPG (Wolff et al., 2008; Witt et al., 2012), and could also be evaluated for efficacy in clinical trials of pediatric brain tumors. Moreover, fingolimod (FTY720), an immunosuppressant agent currently used clinically in the treatment of multiple sclerosis, displays HDAC-inhibiting properties and has been recently shown to reduce the growth of experimental MB (Garner et al., 2018; Perla et al., 2020). Reduced D283 and Daoy cell viability by fingolimod was accompanied by increases in acetylated histone H3 levels, highlighting a role for histone acetylation (Perla et al., 2020). Thus, fingolimod is a new candidate drug for clinical testing in patients with MB. One potential limitation for the clinical use of some HDACis such as panobinostat for brain tumors is poor permeability across the blood–brain barrier (BBB) after oral administration (Rodgers et al., 2020). Novel formulations and drug administration techniques such as convection-enhanced delivery (CED) are emerging strategies to bypass the BBB, one example being MTC110, a water-soluble formulation of panobinostat (Singleton et al., 2018). Pharmacogenomic differences among individual patients pose another challenge for the clinical use of HDACis. Polymorphic enzymes and drug transporters are involved in metabolizing and transporting HDACis, making genotype-specific dose a strategy to reduce the risk of toxicity and avoid suboptimal treatment (Goey et al., 2016). Taken together, the evidence reviewed here strongly provides support for further clinical testing of HDACis as part of the pharmacological treatment available to pediatric brain cancer patients, particularly those with MB or DIPG, tumor types for which there is a larger body of experimental evidence. As the field of therapeutic use of HDACis for the treatment of brain cancer evolves, one can expect the development and testing of more selective HDACis that will target specific HDACs and alter the acetylation status of a relatively small number of substrates, potentially reducing side effects associated with less selective HDACis.

## Author Contributions

All authors listed have made a substantial, direct and intellectual contribution to the work, and approved it for publication.

## Conflict of Interest

The authors declare that the research was conducted in the absence of any commercial or financial relationships that could be construed as a potential conflict of interest.

## References

[B1] Algar E. M. Muscat A. Dagar V. Rickert C. Chow C. W. Biegel J. A. (2009). Imprinted CDKN1C is a tumor suppressor in rhabdoid tumor and activated by restoration of SMARCB1 and histone deacetylase inhibitors. *PLoS One* 4:e4482. 10.1371/journal.pone.0004482 19221586 PMC2637419

[B2] Almeida V. R. Vieira I. A. Buendia M. Brunetto A. T. Gregianin L. J. Brunetto A. L. (2017). Combined treatments with a retinoid receptor agonist and epigenetic modulators in human neuroblastoma cells. *Mol. Neurobiol.* 54 7610–7619. 10.1007/s12035-016-0250-3 27832522

[B3] Anastas J. N. Zee B. M. Kalin J. H. Kim M. Guo R. Alexandrescu S. (2019). Re-programing chromatin with a bifunctional LSD1/HDAC inhibitor induces therapeutic differentiation in DIPG. *Cancer Cell* 36 528–544.e10. 31631026 10.1016/j.ccell.2019.09.005

[B4] Atlasi Y. Stunnenberg H. G. (2017). The interplay of epigenetic marks during stem cell differentiation and development. *Nat. Rev. Genet.* 18 643–658. 10.1038/nrg.2017.57 28804139

[B5] Autin P. Blanquart C. Fradin D. (2019). Epigenetic drugs for cancer and microRNAs: a focus on histone deacetylase inhibitors. *Cancers* 11:1530. 10.3390/cancers11101530 31658720 PMC6827107

[B6] Azzarelli R. Simons B. D. Philpott A. (2018). The developmental origin of brain tumours: a cellular and molecular framework. *Development* 145:dev162693. 10.1242/dev.162693 29759978 PMC6001369

[B7] Bahmad H. F. Elajami M. K. El Zarif T. Bou-Gharios J. Abou-Antoun T. Abou-Kheir W. (2020). Drug repurposing towards targeting cancer stem cells in pediatric brain tumors. *Cancer Metastasis Rev.* 39 127–148. 10.1007/s10555-019-09840-2 31919619

[B8] Bahmad H. F. Poppiti R. J. (2020). Medulloblastoma cancer stem cells: molecular signatures and therapeutic targets. *J. Clin. Pathol.* 73 243–249. 10.1136/jclinpath-2019-206246 32034059

[B9] Bayliss J. Mukherjee P. Lu C. Jain S. U. Chung C. Martinez D. (2016). Lowered H3K27me3 and DNA hypomethylation define poorly prognostic pediatric posterior fossa ependymomas. *Sci. Transl. Med* 8:366ra161. 10.1126/scitranslmed.aah6904 27881822 PMC5123566

[B10] Bhattacharya D. Pomeroy S. L. Pomeranz Krummel D. A. Sengupta S. (2020). Epigenetics and survivorship in pediatric brain tumor patients. *J. Neurooncol.* 10.1007/s11060-020-03535-3 [Epub ahead of print]. 32451770

[B11] Black J. C. Manning A. L. Van Rechem C. Kim J. Ladd B. Cho J. (2013). KDM4A lysine demethylase induces site-specific copy gain and rereplication of regions amplified in tumors. *Cell* 154 541–555. 10.1016/j.cell.2013.06.051 23871696 PMC3832053

[B12] Blattmann C. Oertel S. Thiemann M. Weber K. J. Schmezer P. Zelezny O. (2012). Suberoylanilide hydroxamic acid affects γH2AX expression in osteosarcoma, atypical teratoid rhabdoid tumor and normal tissue cell lines after irradiation. *Strahlenther. Onkol.* 188 168–176. 10.1007/s00066-011-0028-5 22249335

[B13] Bolden J. E. Peart M. J. Johnstone R. W. (2006). Anticancer activities of histone deacetylase inhibitors. *Nat. Rev. Drug Discov.* 5 769–784. 10.1038/nrd2133 16955068

[B14] Buczkowicz P. Hoeman C. Rakopoulos P. Pajovic S. Letourneau L. Dzamba M. (2014). Genomic analysis of diffuse intrinsic pontine gliomas identifies three molecular subgroups and recurrent activating ACVR1 mutations. *Nat. Genet.* 46 451–456. 24705254 10.1038/ng.2936 PMC3997489

[B15] Canettieri G. Di Marcotullio L. Greco A. Coni S. Antonucci L. Infante P. (2010). Histone deacetylase and Cullin3-REN(KCTD11) ubiquitin ligase interplay regulates Hedgehog signalling through Gli acetylation. *Nat. Cell Biol.* 12 132–142. 10.1038/ncb2013 20081843

[B16] Chen C. S. Weng S. C. Tseng P. H. Lin H. P. Chen C. S. (2005). Histone acetylation-independent effect of histone deacetylase inhibitors on Akt through the reshuffling of protein phosphatase 1 complexes. *J. Biol. Chem.* 280 38879–38887. 10.1074/jbc.m505733200 16186112

[B17] Choi S. A. Lee C. Kwak P. A. Park C. K. Wang K. C. Phi J. H. (2019). Histone deacetylase inhibitor panobinostat potentiates the anti-cancer effects of mesenchymal stem cell-based sTRAIL gene therapy against malignant glioma. *Cancer Lett.* 442 161–169. 10.1016/j.canlet.2018.10.012 30367915

[B18] De Smaele E. Di Marcotullio L. Moretti M. Pelloni M. Occhione M. A. Infante P. (2011). Identification and characterization of KCASH2 and KCASH3, 2 novel Cullin3 adaptors suppressing histone deacetylase and Hedgehog activity in medulloblastoma. *Neoplasia* 13 374–385. 21472142 10.1593/neo.101630 PMC3071086

[B19] Dhanyamraju P. K. Holz P. S. Finkernagel F. Fendrich V. Lauth M. (2015). Histone deacetylase 6 represents a novel drug target in the oncogenic Hedgehog signaling pathway. *Mol. Cancer Ther.* 14 727–739. 10.1158/1535-7163.mct-14-0481 25552369

[B20] D’Mello S. R. (2020). Histone deacetylases 1, 2 and 3 in nervous system development. *Curr. Opin. Pharmacol.* 50 74–81. 10.1016/j.coph.2019.11.007 31901696

[B21] Dubuc A. M. Mack S. Unterberger A. Northcott P. A. Taylor M. D. (2012). The epigenetics of brain tumors. *Methods Mol. Biol.* 863 139–153. 10.1007/978-1-61779-612-8_8 22359291

[B22] Ecker J. Oehme I. Mazitschek R. Korshunov A. Kool M. Hielscher T. (2015). Targeting class I histone deacetylase 2 in MYC amplified group 3 medulloblastoma. *Acta Neuropathol. Commun.* 3:22. 10.1186/s40478-015-0201-7 PMC4382927 25853389

[B23] Erkek S. Johann P. D. Finetti M. A. Drosos Y. Chou H. C. Zapatka M. (2019). Comprehensive analysis of chromatin states in atypical teratoid/rhabdoid tumor identifies diverging roles for SWI/SNF and polycomb in gene regulation. *Cancer Cell* 35 95–110.e8. 30595504 10.1016/j.ccell.2018.11.014 PMC6341227

[B24] Falkenberg K. J. Johnstone R. W. (2014). Histone deacetylases and their inhibitors in cancer, neurological diseases and immune disorders. *Nat. Rev. Drug Discov.* 13 673–691. 10.1038/nrd4360 25131830

[B25] Ferretti E. De Smaele E. Miele E. Laneve P. Po A. Pelloni M. (2008). Concerted microRNA control of Hedgehog signalling in cerebellar neuronal progenitor and tumour cells. *EMBO J.* 27 2616–2627. 10.1038/emboj.2008.172 18756266 PMC2567402

[B26] Fouladi M. Park J. R. Stewart C. F. Gilbertson R. J. Schaiquevich P. Sun J. (2010). Pediatric phase I trial and pharmacokinetic study of vorinostat: a Children’s Oncology Group phase I consortium report. *J. Clin. Oncol.* 28 3623–3629. 10.1200/jco.2009.25.9119 20606092 PMC2917318

[B27] Frühwald M. C. Biegel J. A. Bourdeaut F. Roberts C. W. Chi S. N. (2016). Atypical teratoid/rhabdoid tumors-current concepts, advances in biology, and potential future therapies. *Neuro Oncol.* 18 764–778. 10.1093/neuonc/nov264 26755072 PMC4864253

[B28] Frühwald M. C. O’Dorisiom M. S. Dai Z. Rush L. J. Krahe R. Smiraglia D. J. (2001). Aberrant hypermethylation of the major breakpoint cluster region in 17p11.2 in medulloblastomas but not supratentorial PNETs. *Genes Chromosomes Cancer* 30 38–47. 10.1002/1098-2264(2000)9999:9999<::aid-gcc1052>3.0.co;2-s 11107174

[B29] Funato K. Major T. Lewis P. W. Allis C. D. Tabar V. (2014). Use of human embryonic stem cells to model pediatric gliomas with H3.3K27M histone mutation. *Science* 346 1529–1533. 10.1126/science.1253799 25525250 PMC4995593

[B30] Furchert S. E. Lanvers-Kaminsky C. Juürgens H. Jung M. Loidl A. Frühwald M. C. (2007). Inhibitors of histone deacetylases as potential therapeutic tools for high-risk embryonal tumors of the nervous system of childhood. *Int. J. Cancer* 120 1787–1794. 10.1002/ijc.22401 17230517

[B31] Garg N. Vijayakumar T. Bakhshinyan D. Venugopal C. Singh S. K. (2015). MicroRNA regulation of brain tumour initiating cells in central nervous system tumours. *Stem Cells Int.* 2015:141793. 10.1155/2015/141793 PMC4433683 26064134

[B32] Garner E. F. Williams A. P. Stafman L. L. Aye J. M. Mroczek-Musulman E. Moore B. P. (2018). FTY720 decreases tumorigenesis in group 3 medulloblastoma patient-derived xenografts. *Sci. Rep.* 8:6913. 10.1038/s41598-018-25263-5 PMC5932040 29720672

[B33] Geron L. Borges K. S. Andrade A. F. Suazo V. K. Scrideli C. A. Tone L. G. (2015). Antitumour activity of AMG 900 alone or in combination with histone deacetylase inhibitor SaHa on medulloblastoma cell lines. *Neurol. Res.* 37 703–711. 10.1179/1743132815y.0000000048 26000978

[B34] Glozak M. A. Sengupta N. Zhang X. Seto E. (2005). Acetylation and deacetylation of non-histone proteins. *Gene* 363 15–23. 10.1016/j.gene.2005.09.010 16289629

[B35] Goey A. K. Sissung T. M. Peer C. J. Figg W. D. (2016). Pharmacogenomics and histone deacetylase inhibitors. *Pharmacogenomics* 17 1807–1815. 10.2217/pgs-2016-0113 27767376 PMC5558504

[B36] Graham C. Tucker C. Creech J. Favours E. Billups C. A. Liu T. (2006). Evaluation of the antitumor efficacy, pharmacokinetics, and pharmacodynamics of the histone deacetylase inhibitor depsipeptide in childhood cancer models *in vivo*. *Clin. Cancer Res.* 12 223–234. 10.1158/1078-0432.ccr-05-1225 16397046

[B37] Grasso C. S. Tang Y. Truffaux N. Berlow N. E. Liu L. Debily M. A. (2015). Functionally defined therapeutic targets in diffuse intrinsic pontine glioma. *Nat. Med.* 21 555–559. 25939062 10.1038/nm.3855 PMC4862411

[B38] Gudas L. J. (2013). Retinoids induce stem cell differentiation via epigenetic changes. *Semin. Cell Dev. Biol.* 24 701–705. 10.1016/j.semcdb.2013.08.002 23973942 PMC3849227

[B39] Guerreiro Stucklin A. S. Ramaswamy V. Daniels C. Taylor M. D. (2018). Review of molecular classification and treatment implications of pediatric brain tumors. *Curr. Opin. Pediatr.* 30 3–9. 10.1097/mop.0000000000000562 29315108

[B40] Häcker S. Dittrich A. Mohr A. Schweitzer T. Rutkowski S. Krauss J. (2009). Histone deacetylase inhibitors cooperate with IFN-gamma to restore caspase-8 expression and overcome TRAIL resistance in cancers with silencing of caspase-8. *Oncogene* 28 3097–3110. 10.1038/onc.2009.161 19597472

[B41] Häcker S. Karl S. Mader I. Cristofanon S. Schweitzer T. Krauss J. (2011). Histone deacetylase inhibitors prime medulloblastoma cells for chemotherapy-induced apoptosis by enhancing p53-dependent Bax activation. *Oncogene* 30 2275–2281. 10.1038/onc.2010.599 21562496

[B42] Hashizume R. (2017). Epigenetic targeted therapy for diffuse intrinsic pontine glioma. *Neurol. Med. Chir.* 57 331–342. 10.2176/nmc.ra.2017-0018 28592748 PMC5566706

[B43] Hassell K. N. (2019). Histone deacetylases and their inhibitors in cancer epigenetics. *Diseases* 7:E57. 10.3390/diseases7040057 PMC6955926 31683808

[B44] Hellwig M. Merk D. J. Lutz B. Schüller U. (2019). Preferential sensitivity to HDAC inhibitors in tumors with CREBBP mutation. *Cancer Gene Ther.* 27 294–300. 10.1038/s41417-019-0099-5 31068675

[B45] Hennika T. Hu G. Olaciregui N. G. Barton K. L. Ehteda A. Chitranjan A. (2017). Pre-clinical study of panobinostat in xenograft and genetically engineered murine diffuse intrinsic pontine glioma models. *PLoS One* 12:e0169485. 10.1371/journal.pone.0169485 28052119 PMC5215670

[B46] Hoffman M. M. Zylla J. S. Bhattacharya S. Calar K. Hartman T. W. Bhardwaj R. D. (2020). Analysis of dual class I histone deacetylase and lysine demethylase inhibitor domatinostat (4SC-202) on growth and cellular and genomic landscape of atypical teratoid/rhabdoid. *Cancers* 12:756. 10.3390/cancers12030756 32210076 PMC7140080

[B47] Huether R. Dong L. Chen X. Wu G. Parker M. Wei L. (2014). The landscape of somatic mutations in epigenetic regulators across 1,000 paediatric cancer genomes. *Nat. Commun.* 5:3630. 10.1038/ncomms4630 PMC4119022 24710217

[B48] Jaboin J. Wild J. Hamidi H. Khanna C. Kim C. J. Robey R. (2002). MS-27-275, an inhibitor of histone deacetylase, has marked in vitro and in vivo antitumor activity against pediatric solid tumors. *Cancer Res.* 62 6108–6115. 12414635

[B49] Jaeger M. Ghisleni E. C. Cardoso P. S. Siniglaglia M. Falcon T. Brunetto A. T. (2020). HDAC and MAPK/ERK inhibitors cooperate to reduce viability and stemness in medulloblastoma. *J. Mol. Neurosci.* 70 981–992. 10.1007/s12031-020-01505-y 32056089

[B50] Jaeger M. Ghisleni E. C. Fratini L. Brunetto A. L. Gregianin L. J. Brunetto A. T. (2016). Viability of D283 medulloblastoma cells treated with a histone deacetylase inhibitor combined with bombesin receptor antagonists. *Childs Nerv. Syst.* 32 61–64. 10.1007/s00381-015-2963-4 26590027

[B51] Jain S. U. Do T. J. Lund P. J. Rashoff A. Q. Diehl K. L. Cieslik M. (2019). PFA ependymoma-associated protein EZHIP inhibits PRC2 activity through a H3 K27M-like mechanism. *Nat. Commun.* 10:2146. 10.1038/s41467-019-09981-6 PMC6513997 31086175

[B52] Johann P. D. (2020). Invited Review: dysregulation of chromatin remodellers in paediatric brain tumours - SMARCB1 and beyond. *Neuropathol. Appl. Neurobiol.* 46 57–72. 10.1111/nan.12616 32307752

[B53] Johnstone R. W. Licht J. D. (2003). Histone deacetylase inhibitors in cancer therapy: Is transcription the primary target? *Cancer Cell* 4 13–18. 12892709 10.1016/s1535-6108(03)00165-x

[B54] Jones D. T. Jäger N. Kool M. Zichner T. Hutter B. Sultan M. (2012). Dissecting the genomic complexity underlying medulloblastoma. *Nature* 488 100–105. 22832583 10.1038/nature11284 PMC3662966

[B55] Kerl K. Ries D. Unland R. Borchert C. Moreno N. Hasselblatt M. (2013). The histone deacetylase inhibitor SAHA acts in synergism with fenretinide and doxorubicin to control growth of rhabdoid tumor cells. *BMC Cancer* 13:286. 10.1186/1471-2407-13-286 23764045 PMC3693872

[B56] Khaw A. K. Silasudjana M. Banerjee B. Suzuki M. Baskar R. Hande M. P. (2007). Inhibition of telomerase activity and human telomerase reverse transcriptase gene expression by histone deacetylase inhibitor in human brain cancer cells. *Mutat. Res.* 625 134–144. 10.1016/j.mrfmmm.2007.06.005 17669439

[B57] Killick-Cole C. L. Singleton W. G. B. Bienemann A. S. Asby D. J. Wyatt M. J. Boulter L. J. (2017). Repurposing the anti-epileptic drug sodium valproate as an adjuvant treatment for diffuse intrinsic pontine glioma. *PLoS One* 12:e0176855. 10.1371/journal.pone.0176855 28542253 PMC5444593

[B58] Kim K. H. Roberts C. W. (2016). Targeting EZH2 in cancer. *Nat. Med.* 22 128–134. 10.1038/nm.4036 26845405 PMC4918227

[B59] Knipstein J. A. Birks D. K. Donson A. M. Alimova I. Foreman N. K. Vibhakar R. (2012). Histone deacetylase inhibition decreases proliferation and potentiates the effect of ionizing radiation in atypical teratoid/rhabdoid tumor cells. *Neuro Oncol.* 14 175–183. 10.1093/neuonc/nor208 22156471 PMC3266389

[B60] Kouzarides T. (2007). Chromatin modifications and their function. *Cell* 128 693–705. 10.1016/j.cell.2007.02.005 17320507

[B61] Kumar K. S. Sonnemann J. Hong le T. T. Buurman C. Adler F. Maass M. (2007). Histone deacetylase inhibitors, but not vincristine, cooperate with radiotherapy to induce cell death in medulloblastoma. *Anticancer Res.* 27 465–470. 17352268

[B62] Lahm A. Paolini C. Pallaoro M. Nardi M. C. Jones P. Neddermann P. (2007). Unraveling the hidden catalytic activity of vertebrate class IIa histone deacetylases. *Proc. Natl. Acad. Sci. U.S.A.* 104 17335–17340. 10.1073/pnas.0706487104 17956988 PMC2077257

[B63] Lane A. A. Chabner B. A. (2009). Histone deacetylase inhibitors in cancer therapy. *J. Clin. Oncol.* 27 5459–5468. 19826124 10.1200/JCO.2009.22.1291

[B64] Lawlor E. R. Thiele C. J. (2012). Epigenetic changes in pediatric solid tumors: promising new targets. *Clin. Cancer Res.* 18 2768–2779. 10.1158/1078-0432.ccr-11-1921 22589485 PMC3691809

[B65] Lee R. S. Stewart C. Carter S. L. Ambrogio L. Cibulskis K. Sougnez C. (2012). A remarkably simple genome underlies highly malignant pediatric rhabdoid cancers. *J. Clin. Invest.* 122 2983–2988. 10.1172/jci64400 22797305 PMC3408754

[B66] Lee S. J. Lindsey S. Graves B. Yoo S. Olson J. M. Langhans S. A. (2013). Sonic hedgehog-induced histone deacetylase activation is required for cerebellar granule precursor hyperplasia in medulloblastoma. *PLoS One* 8:e71455. 10.1371/journal.pone.0071455 23951168 PMC3739791

[B67] Leichter A. L. Sullivan M. J. Eccles M. R. Chatterjee A. (2017). MicroRNA expression patterns and signalling pathways in the development and progression of childhood solid tumours. *Mol. Cancer* 16:15. 10.1186/s12943-017-0584-0 PMC5248531 28103887

[B68] Li A. M. Dunham C. Tabori U. Carret A. S. McNeely P. D. Johnston D. (2015). EZH2 expression is a prognostic factor in childhood intracranial ependymoma: a Canadian Pediatric Brain Tumor Consortium study. *Cancer* 121 1499–1507. 10.1002/cncr.29198 25586788

[B69] Li X. N. Shu Q. Su J. M. Perlaky L. Blaney S. M. Lau C. C. (2005). Valproic acid induces growth arrest, apoptosis, and senescence in medulloblastomas by increasing histone hyperacetylation and regulating expression of p21Cip1, CDK4, and CMYC. *Mol. Cancer Ther.* 4 1912–1922. 10.1158/1535-7163.mct-05-0184 16373706

[B70] Li Y. Seto E. (2016). HDACs and HDAC inhibitors in cancer development and therapy. *Cold Spring Harb. Perspect. Med.* 6:a026831. 10.1101/cshperspect.a026831 27599530 PMC5046688

[B71] Liu C. Zong H. (2012). Developmental origins of brain tumors. *Curr. Opin. Neurobiol.* 22 844–849. 10.1016/j.conb.2012.04.012 22560511 PMC3432164

[B72] Louis D. N. Perry A. Reifenberger G. von Deimling A. Figarella-Branger D. Cavenee W. K. (2016). The 2016 World Health Organization classification of tumors of the central nervous system: a summary. *Acta Neuropathol.* 131 803–820. 10.1007/s00401-016-1545-1 27157931

[B73] Lue J. K. Prabhu S. A. Liu Y. Gonzalez Y. Verma A. Mundi P. S. (2019). Precision targeting with EZH2 and HDAC inhibitors in epigenetically dysregulated lymphomas. *Clin. Cancer Res.* 25 5271–5283. 10.1158/1078-0432.ccr-18-3989 30979734 PMC6726529

[B74] Mack S. C. Hubert C. G. Miller T. E. Taylor M. D. Rich J. N. (2016). An epigenetic gateway to brain tumor cell identity. *Nat. Neurosci.* 19 10–19. 10.1038/nn.4190 26713744 PMC5568053

[B75] Mack S. C. Taylor M. D. (2009). The genetic and epigenetic basis of ependymoma. *Childs Nerv. Syst.* 25 1195–1201. 10.1007/s00381-009-0928-1 19536551

[B76] Mack S. C. Witt H. Piro R. M. Gu L. Zuyderduyn S. Stütz A. M. (2014). Epigenomic alterations define lethal CIMP-positive ependymomas of infancy. *Nature* 506 445–450. 24553142 10.1038/nature13108 PMC4174313

[B77] Marino A. M. Sofiadis A. Baryawno N. Johnsen J. I. Larsson C. Vukojević V. (2011). Enhanced effects by 4-phenylbutyrate in combination with RTK inhibitors on proliferation in brain tumor cell models. *Biochem. Biophys. Res. Commun.* 411 208–212. 10.1016/j.bbrc.2011.06.141 21726539

[B78] Marshall G. M. Carter D. R. Cheung B. B. Liu T. Mateos M. K. Meyerowitz J. G. (2014). The prenatal origins of cancer. *Nat. Rev. Cancer* 14 277–289. 24599217 10.1038/nrc3679 PMC4041218

[B79] Meel M. H. de Gooijer M. C. Metselaar D. S. Sewing A. C. P. Zwaan K. Waranecki P. (2020). Combined therapy of AXL and HDAC inhibition reverses mesenchymal transition in diffuse intrinsic pontine glioma. *Clin. Cancer Res.* 10.1158/1078-0432.CCR-19-3538 [Epub ahead of print]. 32165429

[B80] Mendez F. M. Núñez F. J. Garcia-Fabiani M. B. Haase S. Carney S. Gauss J. C. (2020). Epigenetic reprogramming and chromatin accessibility in pediatric diffuse intrinsic pontine gliomas: a neural developmental disease. *Neuro Oncol.* 22 195–206. 10.1093/neuonc/noz218 32078691 PMC7032633

[B81] Merchant T. E. (2017). Current clinical challenges in childhood ependymoma: a focused review. *J. Clin. Oncol.* 35 2364–2369. 10.1200/jco.2017.73.1265 28640697

[B82] Milazzo G. Mercatelli D. Di Muzio G. Triboli L. De Rosa P. Perini G. (2020). Histone deacetylases (HDACs): evolution, specificity, role in transcriptional complexes, and pharmacological actionability. *Genes* 11:E556. 10.3390/genes11050556 PMC7288346 32429325

[B83] Milde T. Kleber S. Korshunov A. Witt H. Hielscher T. Koch P. (2011). A novel human high-risk ependymoma stem cell model reveals the differentiation-inducing potential of the histone deacetylase inhibitor Vorinostat. *Acta Neuropathol.* 122 637–650. 10.1007/s00401-011-0866-3 21863243 PMC3889656

[B84] Milde T. Lodrini M. Savelyeva L. Korshunov A. Kool M. Brueckner L. M. (2012). HD-MB03 is a novel Group 3 medulloblastoma model demonstrating sensitivity to histone deacetylase inhibitor treatment. *J. Neurooncol.* 110 335–348. 10.1007/s11060-012-0978-1 23054560

[B85] Milde T. Oehme I. Korshunov A. Kopp-Schneider A. Remke M. Northcott P. (2010). HDAC5 and HDAC9 in medulloblastoma: novel markers for risk stratification and role in tumor cell growth. *Clin. Cancer Res.* 16, 3240–3252. 10.1158/1078-0432.CCR-10-0395 20413433

[B86] Millard C. J. Watson P. J. Fairall L. Schwabe J. W. R. (2017). Targeting class I histone deacetylases in a “complex” environment. *Trends Pharmacol. Sci.* 38 363–377. 10.1016/j.tips.2016.12.006 28139258

[B87] Muscal J. A. Scorsone K. A. Zhang L. Ecsedy J. A. Berg S. L. (2013a). Additive effects of vorinostat and MLN8237 in pediatric leukemia, medulloblastoma, and neuroblastoma cell lines. *Invest. New Drugs* 31 39–45. 10.1007/s10637-012-9831-9 22669335 PMC3655801

[B88] Muscal J. A. Thompson P. A. Horton T. M. Ingle A. M. Ahern C. H. McGovern R. M. (2013b). A phase I trial of vorinostat and bortezomib in children with refractory or recurrent solid tumors: a children’s oncology group phase I consortium study (ADVL0916). *Pediatr. Blood Cancer* 60 390–395. 10.1002/pbc.24271 22887890 PMC3511610

[B89] Muscat A. Popovski D. Jayasekara W. S. Rossello F. J. Ferguson M. Marini K. D. (2016). Low-dose histone deacetylase inhibitor treatment leads to tumor growth arrest and multi-lineage differentiation of malignant rhabdoid tumors. *Clin. Cancer Res.* 22 3560–3570. 10.1158/1078-0432.ccr-15-2260 26920892

[B90] Nagaraja S. Vitanza N. A. Woo P. J. Taylor K. R. Liu F. Zhang L. (2017). Transcriptional dependencies in diffuse intrinsic pontine glioma. *Cancer Cell* 31 635–652.e6. 28434841 10.1016/j.ccell.2017.03.011 PMC5462626

[B91] Nör C. de Farias C. B. Abujamra A. L. Schwartsmann G. Brunetto A. L. Roesler R. (2011). The histone deacetylase inhibitor sodium butyrate in combination with brain-derived neurotrophic factor reduces the viability of DAOY human medulloblastoma cells. *Childs Nerv. Syst.* 27 897–901. 10.1007/s00381-011-1439-4 21560052

[B92] Nör C. Sassi F. A. de Farias C. B. Schwartsmann G. Abujamra A. L. Lenz G. (2013). The histone deacetylase inhibitor sodium butyrate promotes cell death and differentiation and reduces neurosphere formation in human medulloblastoma cells. *Mol. Neurobiol.* 48 533–543. 10.1007/s12035-013-8441-7 23516101

[B93] Northcott P. A. Nakahara Y. Wu X. Feuk L. Ellison D. W. Croul S. (2009). Multiple recurrent genetic events converge on control of histone lysine methylation in medulloblastoma. *Nat. Genet.* 41 465–472. 10.1038/ng.336 19270706 PMC4454371

[B94] Northcott P. A. Shih D. J. Peacock J. Garzia L. Morrissy A. S. Zichner T. (2012). Subgroup-specific structural variation across 1,000 medulloblastoma genomes. *Nature* 488 49–56. 22832581 10.1038/nature11327 PMC3683624

[B95] Ostrom Q. T. Gittleman H. Xu J. Kromer C. Wolinsky Y. Kruchko C. (2016). CBTRUS statistical report: primary brain and other central nervous system tumors diagnosed in the United States in 2009-2013. *Neuro Oncol.* 18(Suppl._5), v1–v75. 10.1093/neuonc/now207 28475809 PMC8483569

[B96] Pak E. MacKenzie E. L. Zhao X. Pazyra-Murphy M. F. Park P. M. C. Wu L. (2019). A large-scale drug screen identifies selective inhibitors of class I HDACs as a potential therapeutic option for SHH medulloblastoma. *Neuro Oncol.* 21 1150–1163. 10.1093/neuonc/noz089 31111916 PMC7594547

[B97] Pal S. Kozono D. Yang X. Fendler W. Fitts W. Ni J. (2018). Dual HDAC and PI3K inhibition abrogates NFκB- and FOXM1-mediated DNA damage response to radiosensitize pediatric high-grade gliomas. *Cancer Res.* 78 4007–4021. 10.1158/0008-5472.can-17-3691 29760046 PMC6294442

[B98] Park P. C. Taylor M. D. Mainprize T. G. Becker L. E. Ho M. Dura W. T. (2003). Transcriptional profiling of medulloblastoma in children. *J. Neurosurg.* 99 534–541. 10.3171/jns.2003.99.3.0534 12959442

[B99] Parsons D. W. Li M. Zhang X. Jones S. Leary R. J. Lin J. C. (2011). The genetic landscape of the childhood cancer medulloblastoma. *Science* 331 435–439. 21163964 10.1126/science.1198056 PMC3110744

[B100] Pei Y. Liu K. W. Wang J. Garancher A. Tao R. Esparza L. A. (2016). HDAC and PI3K antagonists cooperate to inhibit growth of MYC-driven medulloblastoma. *Cancer Cell* 29 311–323. 10.1016/j.ccell.2016.02.011 26977882 PMC4794752

[B101] Perla A. S. Fratini L. Cardoso P. S. de Farias C. B. da Cunha Jaeger M. Roesler R. (2020). Fingolimod (FTY720) reduces viability and survival and increases histone H3 acetylation in medulloblastoma cells. *Pediatr. Hematol. Oncol.* 37 170–175. 10.1080/08880018.2019.1699213 31826690

[B102] Pezuk J. A. Salomão K. B. Baroni M. Pereira C. A. Geron L. Brassesco M. S. (2019). Aberrantly expressed microRNAs and their implications in childhood central nervous system tumors. *Cancer Metastasis Rev.* 38 813–828. 10.1007/s10555-019-09820-6 31797180

[B103] Phi J. H. Choi S. A. Kwak P. A. Lee J. Y. Wang K. C. Hwang D. W. (2017). Panobinostat, a histone deacetylase inhibitor, suppresses leptomeningeal seeding in a medulloblastoma animal model. *Oncotarget* 8 56747–56757. 10.18632/oncotarget.18132 28915627 PMC5593598

[B104] Ramsawhook A. Lewis L. Coyle B. Ruzov A. (2017). Medulloblastoma and ependymoma cells display increased levels of 5-carboxylcytosine and elevated TET1 expression. *Clin. Epigenetics* 9:18. 10.1186/s13148-016-0306-2 PMC5307644 28228863

[B105] Ribatti D. Tamma R. (2020). Epigenetic control of tumor angiogenesis. *Microcirculation* 27:e12602. 10.1111/micc.12602 31863494

[B106] Rodgers L. T. Lester McCully C. M. Odabas A. Cruz R. Peer C. J. Figg W. D. (2020). Characterizing the pharmacokinetics of panobinostat in a non-human primate model for the treatment of diffuse intrinsic pontine glioma. *Cancer Chemother. Pharmacol.* 85 827–830. 10.1007/s00280-019-04021-y 31894347 PMC8459205

[B107] Sanaei M. Kavoosi F. (2019). Histone deacetylases and histone deacetylase inhibitors: molecular mechanisms of action in various cancers. *Adv. Biomed. Res.* 8:63 10.4103/abr.abr_142_19 PMC6839273 31737580

[B108] Schwartzentruber J. Korshunov A. Liu X. Y. Jones D. T. Pfaff E. Jacob K. (2012). Driver mutations in histone H3.3 and chromatin remodelling genes in paediatric glioblastoma. *Nature* 482 226–231. 22286061 10.1038/nature10833

[B109] Scicchitano S. Giordano M. Lucchino V. Montalcini Y. Chiarella E. Aloisio A. (2019). The stem cell-associated transcription co-factor, ZNF521, interacts with GLI1 and GLI2 and enhances the activity of the Sonic hedgehog pathway. *Cell Death Dis.* 10:715. 10.1038/s41419-019-1946-x PMC6763495 31558698

[B110] Shim K. W. Xi G. Farnell B. M. Kim D. S. Tsurubuchi T. Tomita T. (2013). Epigenetic modification after inhibition of IGF-1R signaling in human central nervous system atypical teratoid rhabdoid tumor (AT/RT). *Childs Nerv. Syst.* 29 1245–1251. 10.1007/s00381-013-2087-7 23624780

[B111] Sin-Chan P. Huang A. (2014). DNMTs as potential therapeutic targets in high-risk pediatric embryonal brain tumors. *Expert Opin. Ther. Targets* 18 1103–1107. 10.1517/14728222.2014.938052 25142793

[B112] Singleton W. G. B. Bienemann A. S. Woolley M. Johnson D. Lewis O. Wyatt M. J. (2018). The distribution, clearance, and brainstem toxicity of panobinostat administered by convection-enhanced delivery. *J. Neurosurg. Pediatr.* 22, 288–296. 10.3171/2018.2.PEDS17663 29856296

[B113] Sonnemann J. Kumar K. S. Heesch S. Müller C. Hartwig C. Maass M. (2006). Histone deacetylase inhibitors induce cell death and enhance the susceptibility to ionizing radiation, etoposide, and TRAIL in medulloblastoma cells. *Int. J. Oncol.* 28 755–766. 16465382

[B114] Sonnemann J. Trommer N. Becker S. Wittig S. Grauel D. Palani C. D. (2012). Histone deacetylase inhibitor-mediated sensitization to TRAIL-induced apoptosis in childhood malignancies is not associated with upregulation of TRAIL receptor expression, but with potentiated caspase-8 activation. *Cancer Biol. Ther.* 13 417–424. 10.4161/cbt.19293 22313685

[B115] Spiller S. E. Ditzler S. H. Pullar B. J. Olson J. M. (2008). Response of preclinical medulloblastoma models to combination therapy with 13-cis retinoic acid and suberoylanilide hydroxamic acid (SAHA). *J. Neurooncol.* 87 133–141. 10.1007/s11060-007-9505-1 18060600

[B116] Sredni S. T. Halpern A. L. Hamm C. A. Bonaldo M. F. Tomita T. (2013). Histone deacetylases expression in atypical teratoid rhabdoid tumors. *Childs Nerv. Syst.* 29 5–9. 10.1007/s00381-012-1965-8 23143003

[B117] Surani M. A. Hayashi K. Hajkova P. (2007). Genetic and epigenetic regulators of pluripotency. *Cell* 128 747–762. 10.1016/j.cell.2007.02.010 17320511

[B118] Tang Y. Yacoub A. Hamed H. A. Poklepovic A. Tye G. Grant S. (2012). Sorafenib and HDAC inhibitors synergize to kill CNS tumor cells. *Cancer Biol. Ther.* 13 567–574. 10.4161/cbt.19771 22406992 PMC3679096

[B119] Taylor M. D. Poppleton H. Fuller C. Su X. Liu Y. Jensen P. (2005). Radial glia cells are candidate stem cells of ependymoma. *Cancer Cell* 8 323–335. 10.1016/j.ccr.2005.09.001 16226707

[B120] Thiemann M. Oertel S. Ehemann V. Weichert W. Stenzinger A. Bischof M. (2012). *In vivo* efficacy of the histone deacetylase inhibitor suberoylanilide hydroxamic acid in combination with radiotherapy in a malignant rhabdoid tumor mouse model. *Radiat. Oncol.* 7:52. 10.1186/1748-717x-7-52 22458853 PMC3342162

[B121] Tsikitis M. Zhang Z. Edelman W. Zagzag D. Kalpana G. V. (2005). Genetic ablation of Cyclin D1 abrogates genesis of rhabdoid tumors resulting from Ini1 loss. *Proc. Natl. Acad. Sci. U.S.A.* 102 12129–12134. 10.1073/pnas.0505300102 16099835 PMC1189334

[B122] Urvalek A. M. Gudas L. J. (2014). Retinoic acid and histone deacetylases regulate epigenetic changes in embryonic stem cells. *J. Biol. Chem.* 289 19519–19530. 10.1074/jbc.m114.556555 24821725 PMC4094062

[B123] Vibhakar R. Foltz G. Yoon J. G. Field L. Lee H. Ryu G. Y. (2007). Dickkopf-1 is an epigenetically silenced candidate tumor suppressor gene in medulloblastoma. *Neuro Oncol.* 9 135–144. 10.1215/15228517-2006-038 17329407 PMC1871668

[B124] Visvader J. E. (2011). Cells of origin in cancer. *Nature* 469 314–322. 21248838 10.1038/nature09781

[B125] Waha A. Koch A. Hartmann W. Mack H. Schramm J. Sörensen N. (2004). Analysis of HIC-1 methylation and transcription in human ependymomas. *Int. J. Cancer* 110 542–549. 10.1002/ijc.20165 15122586

[B126] Wang J. Wechsler-Reya R. J. (2014). The role of stem cells and progenitors in the genesis of medulloblastoma. *Exp. Neurol.* 260 69–73. 10.1016/j.expneurol.2012.11.014 23178582 PMC3718859

[B127] Wang X. Lee R. S. Alver B. H. Haswell J. R. Wang S. Mieczkowski J. (2017). SMARCB1-mediated SWI/SNF complex function is essential for enhancer regulation. *Nat. Genet.* 49 289–295. 10.1038/ng.3746 27941797 PMC5285474

[B128] Watanabe M. Adachi S. Matsubara H. Imai T. Yui Y. Mizushima Y. (2009). Induction of autophagy in malignant rhabdoid tumor cells by the histone deacetylase inhibitor FK228 through AIF translocation. *Int. J. Cancer* 124 55–67. 10.1002/ijc.23897 18821579

[B129] Witt O. Milde T. Deubzer H. E. Oehme I. Witt R. Kulozik A. (2012). Phase I/II intra-patient dose escalation study of vorinostat in children with relapsed solid tumor, lymphoma or leukemia. *Klin. Padiatr.* 224 398–403. 10.1055/s-0032-1323692 22915450

[B130] Wolff J. E. Kramm C. Kortmann R. D. Pietsch T. Rutkowski S. Jorch N. (2008). Valproic acid was well tolerated in heavily pretreated pediatric patients with high-grade glioma. *J. Neurooncol.* 90 309–314. 10.1007/s11060-008-9662-x 18679579

[B131] Wu G. Broniscer A. McEachron T. A. Lu C. Paugh B. S. Becksfort J. (2012). Somatic histone H3 alterations in pediatric diffuse intrinsic pontine gliomas and non-brainstem glioblastomas. *Nat. Genet.* 44 251–253. 10.1038/ng.1102 22286216 PMC3288377

[B132] Wu G. Diaz A. K. Paugh B. S. Rankin S. L. Ju B. Li Y. (2014). The genomic landscape of diffuse intrinsic pontine glioma and pediatric non-brainstem high-grade glioma. *Nat. Genet.* 46 444–450. 10.1038/ng.2938 24705251 PMC4056452

[B133] Yuan J. Llamas Luceño N. Sander B. Golas M. M. (2017). Synergistic anti-cancer effects of epigenetic drugs on medulloblastoma cells. *Cell. Oncol.* 40 263–279. 10.1007/s13402-017-0319-7 28429280 PMC13001549

[B134] Zhang H. Zhu D. Zhang Z. Kaluz S. Yu B. Devi N. S. (2020). EZH2 targeting reduces medulloblastoma growth through epigenetic reactivation of the BAI1/p53 tumor suppressor pathway. *Oncogene* 39 1041–1048. 10.1038/s41388-019-1036-7 31582835 PMC7780546

[B135] Zhang S. Gong Z. Oladimeji P. O. Currier D. G. Deng Q. Liu M. (2019). A high-throughput screening identifies histone deacetylase inhibitors as therapeutic agents against medulloblastoma. *Exp. Hematol. Oncol.* 8:30. 10.1186/s40164-019-0153-x PMC6858705 31788346

[B136] Zhao J. Quan H. Xie C. Lou L. (2014). NL-103, a novel dual-targeted inhibitor of histone deacetylases and hedgehog pathway, effectively overcomes vismodegib resistance conferred by Smo mutations. *Pharmacol. Res. Perspect.* 2:e00043. 10.1002/prp2.43 25505589 PMC4186412

